# Critical Care Sedation: Emerging Clinical Considerations and Risks of Volatile Anesthetics for Sedation: A Narrative Review

**DOI:** 10.3390/diseases14040117

**Published:** 2026-03-25

**Authors:** Austin M. Breaux, Garret R. Miller, Harrison D. Cooper, Kristin Nicole Bembenick, Aishwarya Reddy, Shahab Ahmadzadeh, Sahar Shekoohi, Alan D. Kaye

**Affiliations:** 1 School of Medicine, Louisiana State University Health Sciences Center at Shreveport, Shreveport, LA 71103, USA; 2 Department of Anesthesiology, Louisiana State University Health Sciences Center at Shreveport, Shreveport, LA 71103, USA

**Keywords:** volatile anesthetics, sedation, intensive care units, anxiolysis, propofol, sevoflurane, isoflurane

## Abstract

Volatile anesthetics have steadily become more popular in intensive care units for sedation for reasons related to their beneficial pharmacokinetic and pharmacodynamic properties. Common anesthetics such as isoflurane and sevoflurane rapidly reach sedative levels in the body, but they are also rapidly eliminated, allowing for quick recovery. These agents have minimal impact on the liver and kidneys, which makes them attractive options when compared to other agents including opioids, benzodiazepines, ketamine, and propofol. Use of delivery systems like AnaConDa^®^ (Anaesthetic Conserving Device; Sedana Medical AB, Danderyd, Sweden) has enabled providers to easily use these agents in the Intensive Care Unit (ICU). In this regard, they have recently provided additional beneficial consideration during intravenous drug shortages seen during the COVID-19 pandemic and at other times. These agents have shown organ-protective effects in the kidneys and lungs, which may even reduce the total time spent in the ICU. Pharmacodynamically, these anesthetics mediate their effects through central nervous system ion channels to exert analgesic and anxiolytic actions, thereby minimizing effects in the kidneys and lungs. These agents are primarily eliminated via exhalation, which makes them potential options for those with liver or kidney failure. This narrative review examines current efficacy and risks of using volatile anesthetics for sedation in the ICU setting and clinical roles for the future.

## 1. Introduction

Volatile anesthetics have long been used for anesthesia in the operating room, but their use has become more common in inpatient settings such as intensive care units (ICUs). These agents are commonly used for both the induction and the maintenance of general anesthesia in the operating room. Volatile anesthetics are stored as liquids and require the use of vaporizers for inhalation and anesthetic mediated properties. Intravenous sedation in the ICU previously utilized drugs such as propofol, ketamine, midazolam, lorazepam, fentanyl and morphine, but these drugs have long been associated with delirium, central nervous system depression, respiratory depression, hypoxia, and increased time to discharge from the ICU. The operating room has been the typical location for volatile anesthetics; however, recently, there has been an increasing role for use in the inpatient setting. These anesthetics have been shown to provide protection to vital bodily functions in seriously ill patients, such as those in ICUs. In this regard, primary volatile anesthetic elimination is independent of liver or kidney function [1]. This is just one potential benefit of these agents in the ICU setting where there is often some form of multi organ failure.

These anesthetics have the potential to both shorten the duration of ICU visits and cut the associated healthcare costs to patients. These potential benefits would impact both the patients themselves and individual health care teams as a whole. In terms of global prevalence, the use of volatile agents in the ICU remains relatively low but is increasing steadily in certain regions such as Europe. A 2019 national survey in France reported that 21% of ICUs were using volatile anesthetics for sedation [2]. However, the adoption of volatile sedation in ICU settings especially in the United States remains cautious related to emerging evidence of serious safety concerns. These safety concerns have therefore influenced a large amount of research into the potential benefits and risks of these volatile anesthetics. A recent meta-analysis of 21 randomized controlled trials reported a higher mortality risk associated with volatile sedation, particularly sevoflurane, compared to IV sedation, despite the modestly reduced time to extubation [3]. The purpose of this review is to clarify the use of these volatile anesthetics at present and to appreciate pharmacodynamic and pharmacokinetic considerations in several different anesthetics. In this investigation, we discuss various inhaled anesthetics, efficacy, and risks of inhaled sedation in modern ICU practice, and highlight areas requiring further prospective investigation. We will also discuss the potential benefits of these anesthetics in the future. This review evaluates the current literature regarding volatile anesthetics and their current use in the ICU, and it discusses the arguments for and against their use by the writers.

## 2. Current Use of Volatile Anesthetics for Sedation Prevalence

Isoflurane and sevoflurane have become two of the most widely used agents for sedation in the ICU [1]. Typically, Isoflurane has been preferred in the neuro-ICU setting due to its minimal elevation of intracranial pressure and its lack of effect on cardiovascular stability. Sevoflurane allows for faster titration but may pose risks to the patient with long-term use, including metabolic effects related to fluoride ion by-products [2]. Previously, volatile sedation was only used for specific clinical sedations in which patients had failed IV sedation or those with underlying neurological injuries. New devices, such as the Anaesthetic Conserving device (AnaConDa^®^, Anaesthetic Conserving Device; Sedana Medical AB, Danderyd, Sweden), have enabled clinicians to readily access these anesthetics in ICUs and have even been considered for the treatment of specific cases of acute respiratory distress syndrome to reduce delirium [4]. These anesthetic delivery systems became more popular initially during the COVID-19 pandemic due to shortages of intravenous sedatives. Clinicians at the time resorted to these common anesthetics because they are easily used and accessible [1,4].

In patients commonly hospitalized during this time, the use of precise titration allowed physicians to closely monitor patients’ respiratory drive but also limit the amount of opioid exposure, which would lengthen the time to extubation. This highlights the potential for these anesthetics to not only decrease respiratory burden, but also potentially drastically reduce the necessity for opioid analgesia in the ICU.

## 3. Volatile Anesthetics Pharmacodynamics

Volatile anesthetics produce dose-dependent, reversible depression of the central nervous system while also affecting cardiovascular, respiratory, and cerebral physiology. In the brain, these agents reduce the cerebral metabolic rate of oxygen consumption (CMRO_2_), which may confer neuroprotective effects in ischemic states. However, they simultaneously cause cerebral vasodilation, increasing cerebral blood flow in a concentration-dependent manner [5,6]. At lower doses, reduced metabolic demand may offset vasodilatory effects; at higher concentrations, cerebral blood flow may rise sufficiently to increase intracranial pressure, requiring cautious titration in patients with impaired intracranial compliance [5].

## 4. Mechanism of Action

Volatile anesthetics exert their anesthetic and sedative effects through the multimodal modulation of neuronal ion channels via inhalation, utilizing anesthetic reflection devices [7,8]. A principal mechanism involves the potentiation of γ-aminobutyric acid type A (GABA-A) receptors, increasing chloride conductance, and promoting neuronal hyperpolarization, thereby enhancing inhibitory neurotransmission [9]. In parallel, these agents activate two-pore domain potassium (K2P) channels, including TASK and TREK subtypes, stabilizing the resting membrane potential and contributing to immobility and loss of consciousness.

Additionally, volatile anesthetics inhibit N-methyl-D-aspartate (NMDA) receptors, reducing excitatory glutamatergic transmission and attenuating synaptic activity. The combined enhancement of inhibitory pathways and suppression of excitatory signaling produces the characteristic features of general anesthesia, hypnosis, amnesia, analgesia, and immobility in a dose-dependent and reversible manner [9]. The combined effect of these mechanisms produces a reversible, dose-dependent depression of consciousness, along with analgesia, immobility, and amnesia, hallmark features of general anesthesia.

## 5. Systemic Effects

### 5.1. Central Nervous System

For concise side-by-side organ-specific comparisons, refer to Table 1. Volatile anesthetics cause the dose-dependent depression of CNS activity and are associated with a reduction in the cerebral metabolic rate for oxygen (CMRO_2_). This effect may confer neuroprotective benefits in patients with conditions such as traumatic brain injury or ischemic stroke [5]. Volatile anesthetics reduce cerebral metabolic rate and oxygen consumption, which may protect the brain during periods of ischemia or injury. They provide controlled CNS depression, allowing precise sedation while minimizing excitotoxicity and secondary neuronal damage. Studies suggest that these agents may also attenuate neuroinflammation and help preserve cognitive function in critically ill patients [6]. However, these agents can also induce cerebral vasodilation, which may increase intracranial pressure (ICP), particularly in patients with impaired cerebral autoregulation [6]. Arterial carbon dioxide levels play a critical role in modulating these neurophysiologic effects, and controlled hyperventilation resulting in hypocarbia can partially offset anesthetic-induced increases in ICP [10]. As a result, careful titration of anesthetic concentration and meticulous adjustment of ventilatory parameters are required to optimize neurologic outcomes while minimizing adverse effects.

### 5.2. Cardiovascular System

Most volatile anesthetics cause myocardial depression and peripheral vasodilation, leading to hypotension, but each anesthetic has its own qualities and affects the cardiovascular system as well. This means that, before use, the clinician must tailor the anesthetic to achieve appropriate sedation. For example, Isoflurane reduces systemic vascular resistance (SVR) and may reflexively increase heart rate, whereas sevoflurane tends to have a more stable hemodynamic profile [5]. Desflurane can provoke transient sympathetic activation during rapid increases in concentration [11]. Significantly, these drugs reduce myocardial oxygen consumption, which can be advantageous in ischemic heart disease but also necessitate vigilance in hypovolemic or cardiac-compromised patients. Thus, it is important to be able to identify which volatile anesthetic is needed for each patient and their specific conditions.

### 5.3. Respiratory System

All volatile anesthetics exert dose-dependent effects on respiratory function. They depress ventilatory drive, reduce tidal volume, and increase dead space ventilation, which can result in hypercapnia in the absence of mechanical ventilation [10]. These agents also blunt ventilatory responses to hypoxia and hypercapnia, impair mucociliary clearance, and influence airway resistance. Conversely, their bronchodilatory properties can be advantageous in patients with reactive airway disease. The bronchodilatory effects can decrease pulmonary inflammatory responses, which leads to improved gas exchange in ventilated patients. They may attenuate lung injury in patients with acute respiratory distress syndrome or systemic inflammation. These properties contribute to more stable oxygenation and potentially shorter ventilator times [10]. Among the available agents, sevoflurane is the least pungent and least irritating to the airways, making it particularly suitable for inhalational induction and for patients with asthma or other obstructive airway conditions [11]. Given these respiratory effects, close monitoring of gas exchange and ventilatory parameters is essential.

## 6. Renal and Hepatic Effects

Because volatile anesthetics are primarily eliminated via exhalation rather than metabolism, they have minimal nephrotoxic or hepatotoxic effects. This reduces the risk of drug accumulation and organ dysfunction compared with intravenous sedatives. Evidence suggests they may be safer in patients with pre-existing renal or hepatic impairment [12]. Sevoflurane undergoes limited hepatic metabolism, producing inorganic fluoride ions that have theoretical nephrotoxic potential; however, clinical studies have not demonstrated significant renal injury with routine use [12]. Hepatotoxicity is similarly rare with contemporary agents. In contrast, halothane, now largely obsolete, was associated with immune-mediated hepatitis due to reductive metabolism under hypoxic conditions, leading to the generation of reactive metabolites. This complication has not been observed with newer agents such as isoflurane, desflurane, and sevoflurane, which exhibit favorable hepatic safety profiles [5]. For more information, see Table 1.

## 7. Clinical Implications in the ICU

Even though volatile anesthetics and sedation are typically thought of only as being used in the operating room, their role in ICU sedation care is expanding. At present, using inhalation systems like AnaConDa^®^ (Anaesthetic Conserving Device; Sedana Medical AB, Danderyd, Sweden) or MIRUS, agents such as isoflurane and sevoflurane can be administered without intravenous sedatives [7]. Volatile anesthetics are becoming a helpful option in the ICU, especially for patients who need to be on a ventilator for an extended time. Since they work quickly and wear off fast, it is easier for care teams to check brain function and adjust treatment as needed. Some of these anesthetics might also help protect the brain, which is especially important for patients with head injuries or swelling [8].

## 8. Advantages of ICU Sedation

Volatile anesthetics have rapid onset and offset related to solubility properties in blood and tissues, which allows for precise control over the degree of sedation. They are associated with a lower incidence of ICU delirium and withdrawal symptoms in comparison to benzodiazepines and propofol, which are two of the most common sedating agents [13]. Their bronchodilatory effects are beneficial for patients with reactive airway disease. When appropriately titrated, they reduce cerebral metabolism without appreciably increasing intracranial pressure during hyperventilation, making them useful in neurocritical care patients [14].

## 9. Monitoring Requirements

Close monitoring is essential during the use of volatile anesthetics in the ICU. Continuous end-tidal gas monitoring, along with brainwave monitoring, is necessary to assess volatile anesthetic delivery and the depth of anesthesia. Blood pressure and heart rate must be frequently assessed, related to the potential for cardiovascular depressive effects, since many critical care patients have limited cardiopulmonary reserves. Arterial blood gases are critical for evaluating ventilatory adequacy, particularly because ventilatory drive can be affected. In neurocritical patients, ICP and cerebral perfusion pressure (CPP) should be monitored where applicable [10].

## 10. Risks and Considerations

Volatile anesthetics are associated with several important risks, including dose-dependent cardiovascular and respiratory depression, arrhythmogenic potential (notably with the older volatile halothane), and rare but severe idiosyncratic reactions such as malignant hyperthermia and immune-mediated hepatitis [15,16,17]. Neurotoxicity is a particular concern in vulnerable populations. In this regard, animal and human data suggest that prolonged or repeated exposure in infants and children may increase the risk of neurodevelopmental impairment, a point emphasized by the American Heart Association in the 2024 scientific statement on neurodevelopmental outcomes in congenital heart disease [18,19,20].

Though they are most often used in the operating room, volatile anesthetics are finding a growing role in ICUs. Their unique effects make them helpful in providing longer-term sedation and even protecting the brain in certain situations. Still, using them safely in critically ill patients requires careful dosing, close monitoring, and a good understanding of possible side effects, including headaches, which are most commonly reported in the context of occupational exposure to waste anesthetic gases rather than routine post-sedation patient effects.

In addition to physiologic considerations, practical and institutional factors influence the use of volatile anesthetics in the ICU. These agents require specialized delivery devices, scavenging systems, and staff trained in their administration, which may limit availability in some centers. Environmental exposure and occupational safety must also be addressed, as inadequate scavenging can lead to accumulation of waste anesthetic gas. Cost considerations, including equipment acquisition and maintenance, should be weighed against potential benefits such as reduced ICU length of stay, decreased opioid requirements, and improved neurologic assessments. As experience grows, standardized protocols may help optimize safety and utilization in areas in need of improvement.

## 11. Volatile Anesthetics Pharmacokinetics

Volatile anesthetics are increasingly used in ICUs for sedation related to favorable pharmacokinetic properties. Volatile anesthetics such as sevoflurane, isoflurane, and desflurane are administered via inhalation and characterized by rapid onset, minimal systemic metabolism, and clearance via exhalation [18]. Compared to intravenous sedatives, volatile anesthetics offer enhanced titratability and reduced risk of drug accumulation, especially when hepatic or renal function is a concern. Their pharmacokinetic profiles make them especially useful in managing critically ill patients who require prolonged, controlled sedation.

## 12. Absorption and Distribution

Upon inhalation, volatile anesthetics are rapidly absorbed across the alveolar membrane and into the pulmonary capillary bed of the lung. The blood–gas partition coefficient determines how quickly an anesthetic equilibrates between alveoli and blood. A lower coefficient indicates less solubility in blood, enabling a faster rise in alveolar partial pressure, leading to a faster onset. Among the commonly used agents, desflurane (0.42) equilibrates most rapidly, followed by sevoflurane (0.63) and isoflurane (1.4) [18]. This also translates to a more rapid emergence from sedation upon discontinuation of the drug, a critical feature in ICU settings.

The distribution of volatile anesthetics is primarily influenced by lipid solubility, which mediates their uptake into highly perfused tissues such as the brain. These drugs readily cross the blood–brain barrier due to their lipophilic nature, allowing rapid central nervous system penetration [19]. Lipid solubility also contributes to drug accumulation in adipose tissue during prolonged exposure, potentially slowing recovery in obese or critically ill patients with increased fat to muscle ratios [18,19]. Therefore, this factor must be taken into consideration when in use with this type of patient population.

## 13. Metabolism and Biotransformation

In contrast with many intravenous sedatives, volatile anesthetics undergo minimal hepatic metabolism. The favorable hepatic profile of these drugs provides a significant advantage to patients with hepatic dysfunction. For example, sevoflurane is one of the highest hepatically metabolized volatile anesthetics (5%), primarily by CYP2E1, generating inorganic fluoride ions and compound A, both of which have been linked to renal toxicity [20].

Isoflurane and desflurane, in contrast, undergo much lower biotransformation (0.17% and 0.02%, respectively), limiting systemic metabolite exposure and lowering the risk of renal and hepatic toxicity [18,21].

Although CYP2E1 polymorphisms may alter the metabolism of sevoflurane, clinical impacts appear limited due to the low percentage of the drug processed through the hepatic system. Of note, an advantage of volatile anesthetics is that they do not produce systemic metabolites to the extent that intravenous agents such as midazolam or propofol do. These intravenous agents rely on hepatic transformation, and their metabolites may accumulate with prolonged infusion [22].

## 14. Elimination and Clearance

Elimination of volatile anesthetics occurs almost exclusively via exhalation through the lungs, nearly independent of liver or kidney function. The rate of clearance is influenced by agent solubility, the duration of exposure, and the patient’s minute ventilation. Increasing alveolar ventilation enhances the elimination rate by steepening the alveolar-venous gradient, a feature particularly useful in mechanically ventilated ICU patients [18,19]. Desflurane demonstrates the fastest elimination due to its low solubility and lack of significant tissue accumulation. Sevoflurane and isoflurane are eliminated slower, though still predictably. The context-sensitive half-time is a pharmacologic measure of how the duration of infusion affects emergence time; this measure remains relatively stable for these agents compared to IV sedatives, especially in long-duration sedation [21,22].

### ICU Pharmacokinetic Considerations

In critical care settings, the pharmacokinetics of volatile anesthetics are influenced by physiologic, pathologic, and technical variables. Age, hypothermia, and co-administered central nervous system depressants can decrease the minimum alveolar concentration (MAC) requirements of volatile anesthetics, requiring dose reductions [1,19]. In contrast, hyperthermia, stimulant use, and younger age may increase MAC. Obesity and high lipid stores can prolong awakening times due to delayed redistribution of the anesthetic from adipose tissue [18].

Volatile anesthetics offer unique benefits in ICU sedation protocols. Amongst other benefits, they do not induce tachyphylaxis, and there is no evidence of pharmacologic tolerance or withdrawal syndromes with prolonged use [1,22]. Moreover, bedside tools such as end-tidal gas analyzers allow real-time monitoring of drug levels, enhancing precision and safety in sedation management. These features support their role in minimizing oversedation, facilitating rapid neurological assessment, and potentially improving outcomes in mechanically ventilated patients [21,23].

## 15. Operational Considerations for ICU Volatile Sedation

### 15.1. Infrastructure and Environmental Safety

Implementing volatile anesthetics for sedation in the ICU requires paying attention to technical details, environmental safety, and integration into existing ICU workflows.

Although volatile anesthetics have long been administered in the operating room using anesthesia machines that incorporate vaporizers, gas monitoring, and scavenging systems, their use in the ICU became feasible only with the development of ventilator-compatible delivery and reflection devices that allow safe administration during mechanical ventilation. These technologies permit accurate titration of anesthetic concentrations while limiting environmental release, thereby enabling the practical use of volatile anesthetics outside the operating room environment.

Unlike intravenous sedatives, inhaled anesthetics must be delivered through systems that interface with mechanical ventilation and allow for accurate titration while minimizing anesthetic loss. In practice, this requirement has historically limited their use outside the operating room; however, advances in ventilator-compatible delivery and conservation systems have enabled the safe administration of volatile agents in ICU settings. These advances have enabled the expanded use of volatile anesthetics during periods of high demand or intravenous sedative shortages, such as those encountered during the COVID-19 pandemic [4,8].

In the ICU setting, administration requires specialized delivery systems compatible with mechanical ventilation. Anesthetic reflection devices function by adsorbing exhaled volatile anesthetic and returning a substantial portion during subsequent inspiration, thereby reducing agent consumption and environmental release. Systems such as AnaConDa provide passive reflection with external infusion, whereas MIRUS offers automated end-tidal concentration targeting greater technical complexity and cost [7]. Practical considerations include added circuit dead space, need for scavenging infrastructure, staff training, and environmental impact due to greenhouse gas effects. Despite higher implementation costs, advantages such as rapid titratability, minimal organ accumulation, and predictable emergence support their expanding role in critical care sedation [7,8].

The introduction of these anesthetics is not feasible on a case-by-case basis for individual patients. Unlike intravenous sedatives that have been used previously, inhaled volatile anesthetics require trained personnel, appropriate infrastructure, and environmental safeguards. Implementation therefore must be purposefully planned and approved prior to use in patients. It is essential that appropriate devices be available for delivery of these anesthetics but also for their safety of use. Standardized protocols, monitoring standards, and staff education must also be done with appropriate staff to help maintain safe use and monitoring for patients. These anesthetics must only be used within strict guidelines to ensure patient safety, regulation compliance, and workforce protection.

Environmental and occupational exposure safeguards are essential to the use of volatile anesthetics outside the operating room. Appropriate scavenging mechanisms and adequate room ventilation are necessary to prevent leakage of anesthetic gases into unwanted areas. In ICU environments, both passive scavenging systems (which rely on pressure gradients and absorptive canisters) and active scavenging systems (connected to dedicated vacuum disposal lines) may be required depending on room design. In contrast, standard operating rooms typically have built-in active gas evacuation systems integrated into anesthesia machines. Without these controls, unintended staff or patient exposure may occur, particularly in older ICU environments not originally designed for inhaled agents. Modern ICU-compatible delivery approaches are designed to minimize ambient anesthetic gas concentrations and reduce occupational exposure when institutional infrastructure and monitoring standards are met [7,8]. Implementation therefore requires coordination with hospital engineering services and adherence to established safety protocols to ensure compliance with occupational health guidelines.

### 15.2. Staffing, Training, and Interdisciplinary Coordination

Provider education and interdisciplinary collaboration are also essential for safe and effective use. Many ICU clinicians, nurses, and respiratory therapists have limited routine experience administering inhaled anesthetics; therefore, structured training and collaboration with anesthesiology teams are essential to implementation. Initial implementation efforts often rely on anesthesiology support for protocol development and bedside troubleshooting. Clinical competency includes understanding anesthetic delivery principles, ventilator integration, and early recognition of agent-related adverse effects, including cardiovascular depression and other reactions such as malignant hyperthermia [13,14,15,16]. Prior studies evaluating volatile sedation in ICU settings emphasize developing a multidisciplinary protocol to improve safety and consistency of care [8,21].

### 15.3. Workflow and Sedation Management Considerations

In the ICU, volatile anesthetics offer practical advantages related to their rapid titratability and predictable offset. These properties may simplify daily sedation interruptions and neurologic assessments, particularly in patients requiring prolonged mechanical ventilation. Clinical trials and observational studies have demonstrated reduced time to extubation and improved sedation control compared with intravenous agents, although results have varied across patient populations and institutional protocols. These benefits must also be balanced against emerging safety data [3,7,21]. Additionally, the absence of pharmacologic tolerance and withdrawal syndromes distinguishes volatile anesthetics from commonly used intravenous sedatives and may reduce complications associated with long-term sedation [20,21].

The successful incorporation of volatile anesthetics into ICU sedation practice depends on institutional protocols, staff training, and environmental safeguards. In centers where these conditions are met, volatile anesthetics may serve as a valuable adjunct or alternative to intravenous sedation strategies in select patients.

## 16. Discussion

Volatile anesthetics have emerged as a valuable option for long-term sedation in ICU patients, particularly those requiring mechanical ventilation. Their primary advantage lies in their pharmacokinetic profile, low blood–gas solubility, and minimal systemic metabolism, which allows rapid onset and offset, precise titration, and frequent neurologic assessments. These characteristics make volatile anesthetics particularly suitable for critically ill patients, where careful sedation management is crucial.

Beyond effective sedation, these agents confer additional clinical benefits. CNS depression and a reduced cerebral metabolic rate may provide neuroprotection in patients with brain injuries [22]. Unlike traditional intravenous sedatives, volatile anesthetics are excreted predominantly via the lungs, minimizing the risk of renal or hepatic accumulation. They are also associated with reduced delirium, more predictable sedation levels, and minimal issues with tolerance or withdrawal.

However, their use is not without risk. Dose-dependent cardiovascular and respiratory depression, potential neurotoxicity, and the rare but serious risk of malignant hyperthermia require vigilant monitoring and rapid intervention if complications arise. Despite these concerns, current evidence suggests that when carefully managed, volatile anesthetics offer a flexible and potentially safer alternative for long-term ICU sedation, supporting their growing adoption in critical care practice [24]. For more information, see Table 2 (below).

### Future Perspectives

Volatile anesthetics hold significant potential as a versatile option for long-term ICU sedation, but further research is needed to define their optimal clinical role. Future studies should identify patient populations most likely to benefit, such as those with neurological injury or prolonged mechanical ventilation, and establish evidence-based dosing and monitoring protocols [25,26]. Large multicenter trials comparing volatile anesthetics with conventional intravenous sedatives are essential to clarify effects on delirium, cognitive recovery, and ICU outcomes [27]. Integration into sedation guidelines could standardize safe practice, while advances in delivery systems and automated monitoring may improve precision and adoption. Environmental considerations, including their greenhouse gas effects, underscore the need for low-emission technologies and sustainable ICU practices. One perspective that may drive physicians to move toward volatile anesthetics is the potential adverse effects of propofol and opioids including tolerance, delirium, and withdrawal [25,28]. Addressing these areas will refine the clinical application of volatile anesthetics in critical care.

## 17. Conclusions

The efficacy and risks of the use of volatile anesthetics require a thoughtful discussion due to their high usage in the modern era. This ability is largely related to their blood gas solubility and other favorable pharmacokinetic properties. They leave the body almost entirely by exhalation, which makes them attractive in the ICU. These properties also allow for precise titrations of these gases that can be individualized for each patient to provide more effective sedation. These gases are very effective at providing long-term sedation, which has been established previously in the literature. Like any sedative, they require close monitoring, but because of their favorable properties and minimal effects on the body, they should be considered for the sedation of patients in ICUs.

## Figures and Tables

**Table 1:** Systemic Effects of Volatile Anesthetics. Table derived from references [7,8,9,10,11].

System	Key Effects	Clinical Relevance
CNS	↓ CMRO_2_; possible ↑ ICP	Neuroprotection vs. ICP risk
Cardiovascular	↓ SVR; myocardial depression	Hypotension risk
Respiratory	↓ respiratory drive; bronchodilation	Requires ventilation
Renal	Minimal toxicity; fluoride ions (sevoflurane)	Monitor with prolonged use
Hepatic	Rare toxicity	Modern agents generally safe

CMRO_2_ = Cerebral Metabolic Rate of Oxygen, ICP = intracranial pressure, SVR = Systemic Vascular resistance. ↑, increase; ↓, decrease.

**Table 2:** Pharmacokinetic Characteristics of Common Volatile Anesthetics. Table derived from References [18,19,20,21,22].

Drug	MAC	Blood–Gas Partition Coefficient	% Metabolized	Main Metabolic Enzyme	Elimination Route	Onset/Offset
Sevoflurane	1.8	0.63	5	CYP2E1	Pulmonary exhalation	Moderate
Isoflurane	1.17	1.4	0.17	CYP2E1	Pulmonary exhalation	Slower than sevoflurane
Desflurane	6.6	0.42	0.02	Minor (CYP2E1)	Pulmonary exhalation	Fastest

## Data Availability

No new data were created or analyzed in this study.
